# From Pathophysiology to Innovative Therapies in Eye Diseases: A Brief Overview

**DOI:** 10.3390/ijms26178496

**Published:** 2025-09-01

**Authors:** Karolina Kłodnicka, Jacek Januszewski, Hanna Tyc, Aleksandra Michalska, Alicja Forma, Barbara Teresińska, Robert Rejdak, Jacek Baj, Joanna Dolar-Szczasny

**Affiliations:** 1 Department of Forensic Medicine, Medical University of Lublin, Ul. Jaczewskiego 8b, 20-090 Lublin, Poland; klodnickakarolina02@gmail.com (K.K.); ola.michalska2002@interia.pl (A.M.); formaalicja@gmail.com (A.F.); b.teresinska@gmail.com (B.T.); 2 Department of Correct, Clinical, and Imaging Anatomy, Medical University of Lublin, Jaczewskiego 4, 20-090 Lublin, Poland; jacek.januszewski000@gmail.com (J.J.); hannatyc369@gmail.com (H.T.); 3 Department of General and Pediatric Ophthalmology, Medical University of Lublin, Chmielna 1, 20-079 Lublin, Poland; robertrejdak@umlub.pl

**Keywords:** eye diseases, genetic, biochemical, proteomic, cellular and physiological level, innovative therapeutic approaches

## Abstract

Molecular imaging and precision therapies are transforming ophthalmology, enabling earlier and more accurate diagnosis and targeted treatment of sight-threatening diseases. This review focuses on age-related macular degeneration, diabetic retinopathy, glaucoma, and uveitis, examining high-resolution imaging techniques such as optical coherence tomography (OCT), OCT angiography, MALDI-MSI, and spatial transcriptomics. Artificial intelligence supports these methods by improving image interpretation and enabling personalized analysis. The review also discusses therapeutic advances, including gene therapies (e.g., AAV-mediated RPE65 delivery), stem cell-based regenerative approaches, and biologics targeting inflammatory and neovascular processes. Targeted molecular therapies targeting specific signaling pathways, such as MAPK, are also explored. The combination of single-cell transcriptomics, proteomics, and machine learning facilitates the development of personalized treatment strategies. Although these technologies hold enormous potential, their implementation in routine clinical care requires further validation, regulatory approval, and long-term safety assessment. This review highlights the potential and challenges of integrating molecular imaging and advanced therapies in the future of precision ophthalmic medicine.

## 1. Introduction

Eye diseases are a leading cause of visual impairment and blindness worldwide, representing a significant global health burden that is expected to rise with aging populations and increasing prevalence of chronic systemic diseases. According to the World Health Organization (WHO), over 2.2 billion people globally suffer from visual impairment or blindness, and approximately half of these cases could have been prevented or remain unaddressed due to insufficient access to diagnostic and therapeutic resources [1]. Among the most prevalent vision-threatening conditions are age-related macular degeneration (AMD), diabetic retinopathy (DR), glaucoma, and cataracts. These conditions all involve complex molecular and cellular mechanisms such as oxidative stress, inflammation, apoptosis, and genetic dysregulation [2,3]. In recent years, the field of ophthalmology has undergone a transformative shift with the integration of molecular biology, omics technologies, and precision diagnostics. These innovations allow for a deeper understanding of ocular disease pathophysiology at the genetic, proteomic, metabolomic, and cellular levels. For instance, genomic sequencing technologies have identified over 270 genes associated with inherited retinal disorders, facilitating gene-specific diagnostics and therapies [4]. Simultaneously, advances in proteomics and metabolomics have enabled the discovery of disease-specific biomarkers in ocular fluids. These biomarkers allow for earlier, less invasive, and more accurate diagnosis of retinal, inflammatory, and degenerative conditions [5]. Moreover, modernization of molecular imaging has provided spatial context to these findings, linking molecular alterations to anatomical and functional outcomes [6,7,8]. These advancements have not only enhanced diagnostic precision but have also spurred the development of novel therapeutic strategies, including gene therapy, stem cell-based regeneration, and biological agents such as VEGF inhibitors and monoclonal antibodies. Furthermore, nanomedicine and targeted drug delivery systems are revolutionizing treatment paradigms by improving therapeutic efficacy while minimizing systemic side effects [9]. The convergence of multi-omics technologies, artificial intelligence, and molecular pharmacology holds promise for personalized medicine in ophthalmology, tailoring diagnostics and treatments to individual molecular profiles [10]. This review aims to synthesize the current understanding of the molecular pathophysiology of major eye diseases and explore the role of modern diagnostic technologies in shaping the future of ophthalmic care. By bridging mechanistic insights with clinical application, this paper highlights the potential of molecular ophthalmology to redefine the diagnostic and therapeutic landscape of ophthalmology.

## 2. Anatomy of the Eye

The eye is a sensory organ whose that allows us to see the world by receiving and processing the energy of light as it enters the eye [11]. Its complex anatomical structure reflects this role. The eye is composed of structures that vary in their embryonic origin, morphology, and function [12]. The eye is composed of three main layers: the outer fibrous layer (cornea and sclera), the middle vascular layer (ciliary body, choroid, and iris), and the inner neural layer (retina) [13].

The cornea, anterior chamber, iris, lens, and ciliary body constitute the anterior segment of the eye [14]. Light entering the eye is focused by the cornea. The cornea is a transparent, multilayered structure [15]. Both chambers of the anterior cavity contain aqueous humor, a watery fluid that nourishes the cornea and lens [15]. The constant creation and outflow of fluids maintains this pressure [16]. The pathophysiology of glaucoma is directly dependent on structures such as the aqueous outflow angle and the trabecular meshwork, which help maintain normal intraocular pressure [17]. The lens, which helps focus images on the retina and is supported by the ganglia of Zinn, is susceptible to clouding during cataract development [18].

The retina, choroid, sclera, and vitreous humor constitute the posterior segment of the eye [19]. The retina, the deepest layer of the neuroepithelium, which consists of ten separate layers, contains three main types of neuronal photoreceptors (rodes and cones), bipolar cells, and ganglion cells [19]. The optic nerve is made up of the ganglion cells’ axons [20]. Beneath the retina lies the retinal pigment epithelium (RPE), a pigmented layer essential for photoreceptor function, nutrient transport, and phagocytosis of photoreceptor outer segments [21,22]. Dysfunction of the retinal pigment epithelium plays a key role in diseases such as AMD [23].

The RPE rests on Bruch’s membrane, which separates it from the choroid, the layer that supplies oxygen and micronutrients to the outer retina [24]. The sclera is the outermost fibrous tissue that provides mechanical protection and gives shape to the eyeball [25]. The central area of the retina is the macula, centered on the fovea [26]. These areas are responsible for acute central vision, as the macula consists primarily of cones, the photoreceptors responsible for color vision [27]. Degeneration of these areas is characteristic of AMD [27]. The peripheral retina contains a higher density of rods, enabling night vision [28]. At the posterior pole, the axons of the ganglion cells converge to form the optic disk [29]. The optic disk is a key structure in people affected by glaucoma, where progressive concavity and loss of nerve fibers lead to irreversible vision loss [17].

**Figure 1:**
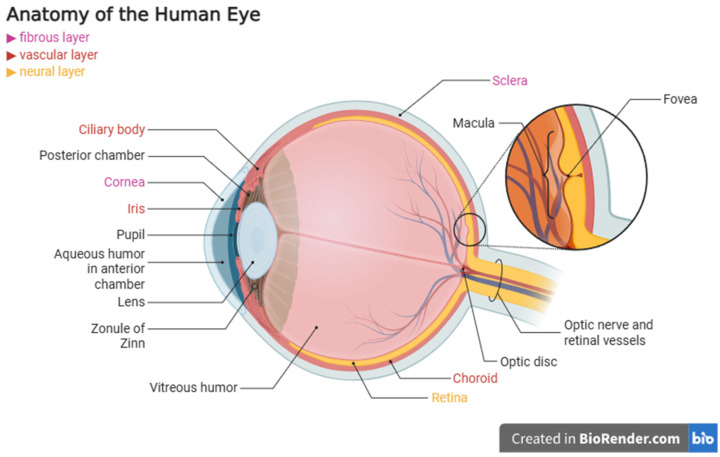
Anatomical Structure of the Healthy Human Eye in the Context of Imaging and Targeted Therapy [11,12,13,14,15,16,17,18,19,20,21,22,23,24,25,26,27,28,29,30,31].

The use of targeted therapies like subretinal gene delivery, stem cell transplantation, or drug systems based on nanoparticles, as well as sophisticated imaging methods like OCT, OCT-A, fluorescein angiography, and molecular imaging, depends on an understanding of the spatial organization of ocular tissues [30]. For example, gene therapy techniques specifically target the RPE or photoreceptors, while spatial transcriptomics enables precise mapping of gene expression across retinal layers [31]. Therefore, knowledge of ocular anatomy improves understanding of the localization of pathological changes, facilitates their early detection, and enhances the accuracy of therapeutic interventions (Figure 1).

## 3. Pathophysiology of Eye Diseases at the Molecular Level—Introduction

Retinal neurons, glial cells, RPE, and vascular endothelial cells are among the many cell types whose proper function determines the integrity of the eye. The eye is a highly specialized and complex sensory organ [32]. When the homeostasis of any of these compartments is disrupted, disease processes can begin or progress, resulting in blindness or visual impairment [33]. Our knowledge of eye disorders has changed dramatically over the last 20 years due to developments in molecular biology and genomics [34]. Complex networks of oxidative, inflammatory, angiogenic, and genetic processes that contribute to their pathophysiology have been uncovered by these findings [35]. In many eye diseases, especially retinal diseases, a multitude of pathological factors have been discovered at the molecular level [36]. These include abnormal angiogenesis, oxidative stress, mitochondrial dysfunction, autophagy dysregulation, and chronic inflammation [37]. These mechanisms often work together to create a self-perpetuating cycle that results in cellular deterioration and tissue destruction [38]. For example, reactive oxygen species (ROS) generated by metabolic stress can induce apoptosis in retinal cells, activate pro-inflammatory signaling pathways (including NF-κB), and damage cellular proteins and DNA [39]. Additionally, genetic predisposition is a significant factor in the onset and progression of disease, particularly in age-related macular degeneration (AMD) and hereditary retinal dystrophies [40]. Variants in some genes can change how angiogenic factors, like VEGF, are controlled, alter inflammatory responses, or impact the structural integrity of photoreceptors [41]. The molecular causes of various retinal diseases will be discussed in this chapter.

### 3.1. AMD—Age-Related Macular Degeneration

**Figure 2:**
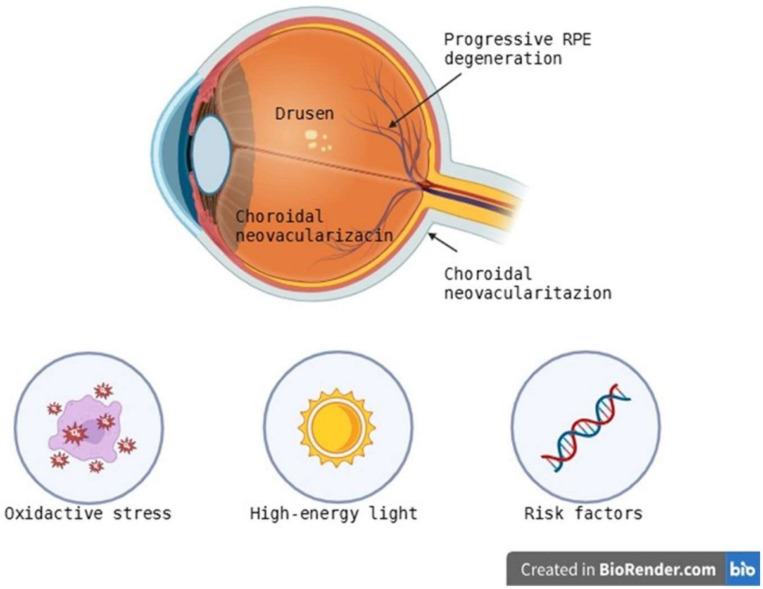
Pathogenesis of Age-Related Macular Degeneration: Structural Changes and Contributing Factors [42,43,44,45,46,47,48,49,50,51,52,53,54,55,56,57,58,59,60,61,62].

AMD, which mainly affects older people, is one of the leading causes of blindness worldwide [42]. The disease’s hallmarks include drusen formation, progressive RPE degeneration, and, in more advanced stages, choroidal neovascularization [43,44]. AMD is further divided into early, intermediate, and advanced phases according to drusen size and pigmentary abnormalities. AMD clinically is divided into wet (neovascular) and dry (atrophic) forms [44]. The etiology of AMD is complex and involves genetic, environmental, and epigenetic factors. An increased risk of AMD is associated with the presence of polymorphisms in genes such as ARMS2, HTRA1, and CFH [45,46,47]. According to a genome-wide association study (GWAS), individuals homozygous for both the ARMS2/HTRA1 risk allele and the CFH risk allele are more than 50 times more likely to develop AMD [47]. The complement system plays a key role in the pathogenesis of AMD through the involvement of other variants in complement-related genes, including C3, CFB, CFI, C2, C9, and VTN [48]. Remarkably, rare high-penetrance mutations in CFH are linked to severe, early-onset illness [49], while a haplotype with deletion in CFHR1 and CFHR3 seems to be protective [50]. According to heritability estimates, 45–70% of AMD risk is due to hereditary factors [51]. Chronic inflammation is implicated, according to an increasing amount of evidence, especially when the alternative complement pathway is dysregulated [52,53]. Uncontrolled activation of CFH and CFI due to impaired regulation by membrane-bound proteins like membrane cofactor protein (MCP) and decay-accelerating factor (DAF) causes membrane attack complex (MAC) deposition at the RPE-Bruch’s membrane (BM)—choriocapillaris interface and structural disruption [53]. In addition, microglial activation and the occurrence of invasive macrophages trigger the release of pro-inflammatory cytokines (TNF-α, IL-1β, IL-6) and chemokines (CCL2) that promote retinal degeneration and disruption of the blood-retinal barrier [54]. Oxidative stress is a key element of AMD pathophysiology (Figure 2). 

High metabolic rate, constant exposure to light, and an oxygen-rich environment make the retina particularly susceptible to the generation of reactive oxygen species (ROS) [55,56]. The macula has the ability to absorb high-energy light to support central vision and is therefore subjected to significant photooxidative stress [56]. When lipofuscin accumulates in retinal pigment epithelial cells, lysosomal degradation is hindered and oxidative damage is exacerbated [57]. Blue light and ultraviolet (UV) exposure intensify these effects, resulting in mitochondrial dysfunction, decreased mitophagy, DNA damage, and ultimately retinal pigment epithelial apoptosis [58]. A factor that includes hypoxia in neovascular (wet) AMD, the transcription factor 1 alpha (HIF-1α) mediates the hypoxia-induced synthesis of vascular endothelial growth factor A (VEGF-A) [59,60,61]. In the macula, VEGF-A promotes the development of immature choroidal neovascular arteries, which results in exudation, scar fibrosis, and bleeding [60,61]. Anti-VEGF drugs are currently considered the standard of care because they prevent neovascularization and reduce fluid accumulation, but treatment resistance and intravitreal injection burden are significant barriers [62].

### 3.2. Diabetic Retinopathy

**Figure 3:**
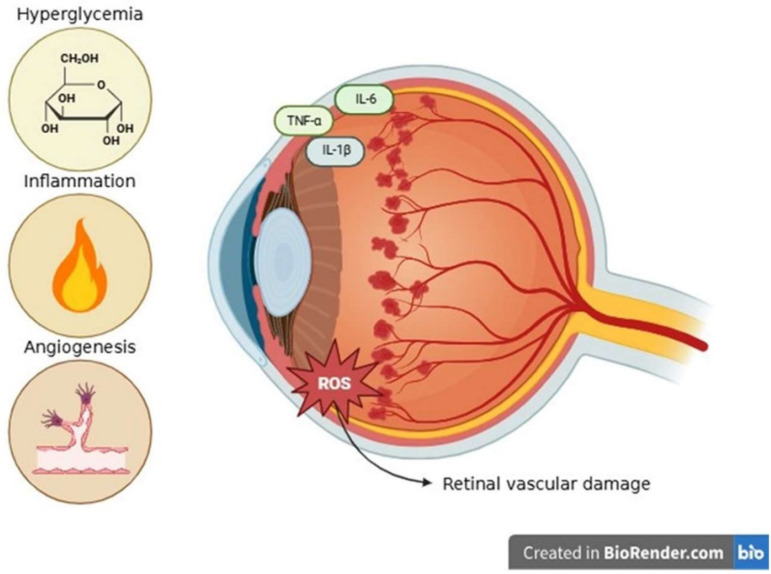
Pathophysiological Mechanisms in Diabetic Retinopathy: From Hyperglycemia to Vascular Damage [63,64,65,66,67,68,69,70,71,72,73,74,75,76,77,78].

Diabetic retinopathy (DR), a chronic microvascular disease primarily caused by persistent hyperglycemia and resulting in progressive retinal vascular damage, is associated with both type 1 and type 2 diabetes [63,64]. The multifactorial pathophysiology of DR includes a complex interplay among oxidative, inflammatory, angiogenic, and metabolic processes [64]. Chronic hyperglycemia induces the accumulation of advanced glycation and end-products (AGEs), and non-enzymatic glycation of proteins interacts with their receptor RAGE to produce pro-inflammatory signaling and oxidative stress, which ultimately results in pericyte loss and vascular dysfunction [65,66,67]. The polyol pathway, which uses aldose reductase to convert excess glucose to sorbitol, consumes NADPH, reduces glutathione regeneration, and increases osmotic stress, leading to damage of endothelial and pericyte cells [68]. The hexosamine biosynthesis pathway alters gene expression through O-linked β-N-acetylglucosaminylation (O-GlcNAcylation), which promotes inflammation, apoptosis, and fibrosis [69]. The protein kinase C (PKC) pathway, activated by hyperglycemia-induced diacylglycerol accumulation, stimulates NADPH oxidase activity, thereby increasing ROS production [70]. These metabolic abnormalities result in excessive generation of ROS and damage to retinal endothelial cells and pericytes, as well as reduced mitochondrial activity [71,72]. ROS also disrupts the blood-retinal barrier, leading to neurovascular dysfunction. When microglia and infiltrating macrophages are activated by oxidative and metabolic stress, they release pro-inflammatory cytokines such as TNF-α, IL-6, and IL-1β [73]. These cytokines promote leukostasis, vascular leakage, and macular edema in association with adhesion molecules such as ICAM-1 and VCAM-1 [74]. The development of DR, and, in particular, the proliferative stages, is influenced by increased intraocular concentrations of inflammatory mediators [75]. VEGF is a key element of pathological neovascularization, which is upregulated in chronic retinal hypoxia [76]. In addition to fibrovascular proliferation, VEGF promotes the formation of new blood vessels, which are delicate and prone to leak into the vitreous body [77]. These processes cause vitreoretinal stress in severe DR, which can lead to retinal detachment, a major cause of irreversible vision loss [78]. In summary, DR is caused by a complex interaction between oxidative stress, inflammation, angiogenesis, and metabolic dysfunction, culminating in progressive neovascular degeneration and vision loss (Figure 3).

### 3.3. Glaucoma

**Figure 4:**
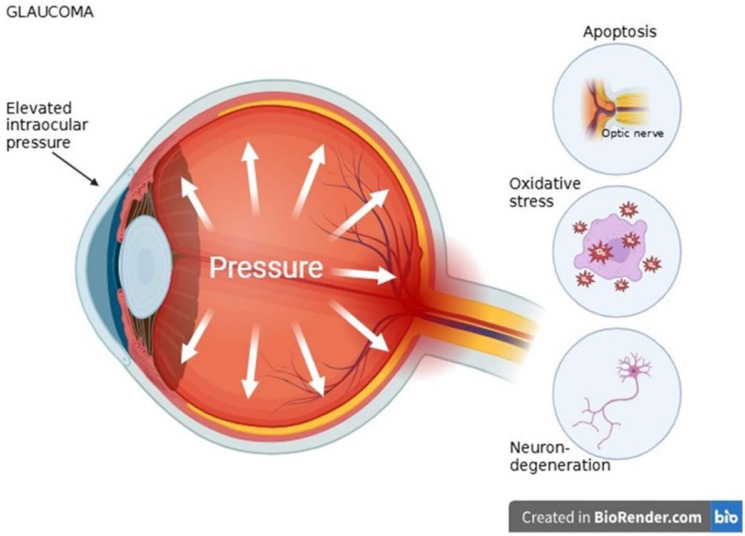
Pathophysiological Changes in Glaucoma: From Intraocular Pressure to Neurodegeneration [79,80,81,82,83,84,85,86,87,88,89,90,91,92,93,94,95].

Elevated intraocular pressure (IOP) has been found to be the only modifiable risk factor validated in large clinical trials for glaucoma, a diverse group of disorders characterized by irreversible vision loss due to progressive degeneration of retinal ganglion cells (RGCs). However, not all patients have elevated IOP [79]. Even with successful IOP control, a sizable portion of patients still experience RCG loss and disease progression, underscoring the significance of additional pathogenic mechanisms beyond IOP elevation, despite the paramount role of IOP reduction in glaucoma management [80]. RCGs are important neurons that transmit visual information to various brain regions, and the optic nerve is composed of their axons [81]. Apoptosis is the main mechanism of RCG death in glaucoma and occurs via two major pathways: extrinsic (death receptor) and intrinsic (mitochondrial) cascades. At the last stage of apoptosis execution, both pathways converge [82]. The BCL-2 family proteins, which regulate mitochondrial outer membrane permeabilization (MOMP) through effectors like BAX and BAK, intricately regulate the intrinsic apoptotic pathway [83]. In glaucoma, RCG apoptosis involves three key intrinsic phases: the balance of pro- and anti-apoptotic BCL-2 proteins, MOMP induction, and subsequent caspase activation. Since irreversible mitochondrial damage makes targeting late-stage caspase activation unlikely to provide neuroprotection, earlier intervention points within this pathway may be more promising therapeutic targets [84]. In addition, signaling pathways such as MAPK/JNK and death receptor ligands such as TNF-α and Fas have been implicated in RCG apoptosis [85]. One of the main causes of glaucomatous degeneration is oxidative stress, which is caused by an imbalance between ROS production and antioxidant defense. The trabecular meshwork (TM) is particularly susceptible to oxidative damage due to its anatomical location and weaker antioxidant mechanisms [86]. The redox homeostasis of the aqueous humor can be disrupted by excess ROS production, which can result in increased outflow resistance, impairing the TM [87]. Glutathione peroxidase, catalase, and superoxide dismutase are key antioxidant enzymes whose activity is reduced and ROS production is increased when mitochondrial dysfunction occurs [88]. RGCs and normal aqueous humor outflow are progressively impaired by aging and genetic polymorphisms. This alters the antioxidant response and increases intraocular pressure [89]. Neurodegeneration is a process that continues even after IOP normalization. Pharmacological treatments such as brimonidine and minocycline have also been shown to increase the activity of the antiapoptotic protein Bcl-2, providing additional neuroprotection [90,91]. Therefore, many studies have focused on neuroprotective techniques that target IOP-independent processes. The activity of endogenous protective factors, such as the heat shock proteins Hsp70 and Hsp27, is necessary for maintaining RGC homeostasis. Heat shock proteins regulate protein folding, reduce oxidative stress, inflammation, and apoptosis. Hsp70 gene polymorphisms have been associated with an increased incidence of primary open-angle glaucoma [92]. These pathological processes are closely linked. Mitochondrial dysfunction and associated ROS accumulation are triggered by oxidative stress, and apoptosis signaling cascades are triggered by mitochondrial dysfunction. Additionally, impaired neuroprotective responses render RGCs even more susceptible to damage [93]. Feedback loops involving oxidative damage, apoptosis, and glial-mediated neuroinflammation perpetuate a vicious cycle of progressive neurodegeneration [94]. Targeted therapies that go beyond lowering intraocular pressure and include molecular neuroprotection are essential to halt the progression of glaucoma [90]. Early diagnosis and individual treatment plans can be more easily proposed by the presence of biomarkers of oxidative stress, such as ROS levels, apoptosis indices, and mitochondrial health indices (Figure 4) [95].

### 3.4. Cataract

Opacities in the lens of the eye that prevent light from reaching the retina are known as cataracts, and they cause progressive visual impairment. According to their anatomical location within the lens, cataracts can be categorized as cortical, nuclear, or mixed, and the majority are age-related [96]. The highly ordered and densely packed arrangement of crystallin proteins—specifically: α-, β-, and γ-crystallins within the lens fiber cells, which are special in that they lack most organelles and nuclei, making them metabolically limited and unable to turn over proteins, is essential for preserving lens transparency and proper refractive function [97,98,99]. As a result, these proteins experience continuous post-translational modifications over time, including oxidation, glycation, truncation, and deamidation, which can lead to structural instability and promote aggregation [100]. This progressive loss of proteostasis is the basic process by which cataracts arise. An important factor in this process is oxidative stress. Protein oxidation and aggregation are accelerated by ROS, which can be produced endogenously or in response to environmental factors like UV radiation, cigarette smoke, and systemic diseases like diabetes mellitus [101,102,103,104,105]. Lens proteins are especially vulnerable to oxidative degradation, especially γ-crystallins. For instance, by encouraging dimerization and aggregation formation through the oxidation of crucial cysteine residues like Cys20, γS-crystallin may further destabilize nearby crystallins [106,107]. The oxidation of methionine residues also impairs the chaperone activity of α-crystallins, which is essential for maintaining lens protein solubility. However, enzymes such as methionine sulfoxide reductase A (MsrA) can partially restore this function [108]. Oxidative alterations in advanced cataracts can impact over 50% of methionine residues and over 90% of cysteine residues, significantly impairing the structural integrity and solubility of crystallins [106]. The lens’s natural antioxidant defense system, which includes enzymes such as glutathione peroxidase (GPX), catalase (CAT), peroxiredoxins (PRDXs), and superoxide dismutase (SOD), typically functions to neutralize ROS and maintain redox equilibrium [109].

**Figure 5:**
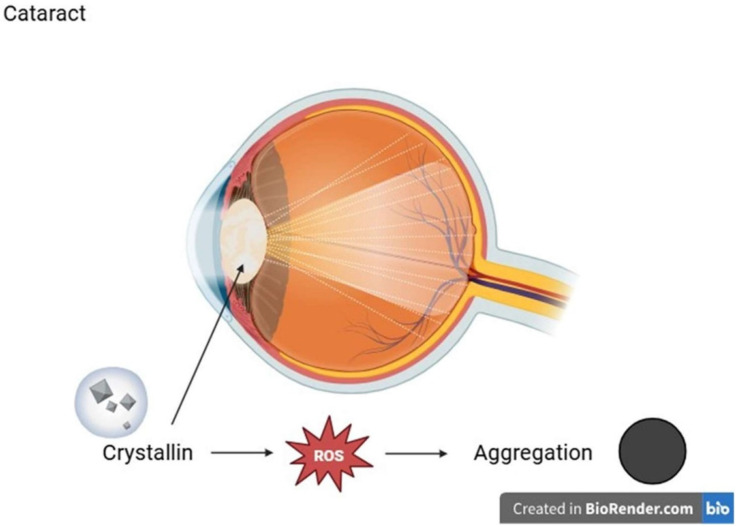
Oxidative Stress and Protein Aggregation in Cataract Formation [96,97,98,99,100,101,102,103,104,105,106,107,108,109,110,111,112].

However, as the body ages, glutathione (GSH) levels decrease, GPX activity is impaired, and free iron levels and lipid peroxidation increase. These biochemical alterations fulfill key criteria of ferroptosis [110,111,112,113,114]. As a result of these processes, oxidized and cross-linked proteins accumulate, which become insoluble even under reducing conditions, which is strongly correlated with the severity of cataract [112]. All these molecular findings reinforce the concept that oxidative damage to crystallin proteins is a central driver of cataract formation. Therefore, maintaining redox homeostasis and increasing antioxidant capacity are important therapeutic goals to delay or avoid the development of cataract (Figure 5).

### 3.5. Inflammatory and Autoimmune Diseases

**Figure 6:**
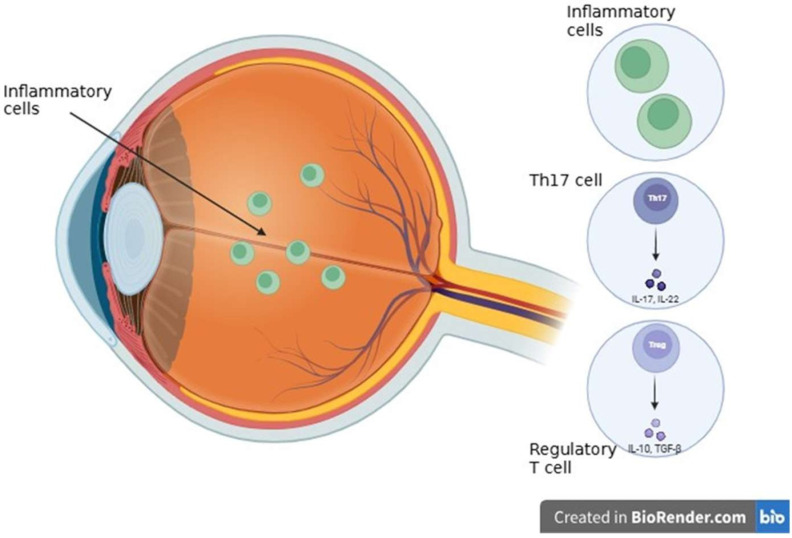
Immune Cell Involvement in Ocular Inflammation [115,116,117,118,119,120,121,122,123,124,125].

Autoimmune uveitis represents a multifaceted interaction between systemic immune dysregulation and local intraocular inflammation [115]. Under physiological conditions, the eye is recognized as an immune-privileged organ, primarily due to the structural integrity of the blood-retina barrier (BRB) and the presence of a precisely regulated immunosuppressive microenvironment [116]. Immune regulation is a process essential for maintaining retinal homeostasis and preventing immune-mediated damage to the highly sensitive and minimally regenerating neural tissue [116]. Disruption of the retinal physical barriers compromises this immune-privileged status and causes retinal disease due to increased BRB permeability and associated higher ocular capillary hydrostatic pressure [117]. The ocular microenvironment temporarily suspends immune privilege to allow an effective inflammatory response, but rapidly initiates mechanisms to restore immunosuppression even before peak retinal inflammation [118]. The dynamic regulation of aqueous humor immunosuppressive properties and the Anterior Chamber-Associated Immune Deviation (ACAID) indicates an instinctive attempt of the eye to balance immune activation with preservation of tissue integrity during uveitis [118]. In the case of uveitis, the BRB is damaged by systemic autoimmune processes or local inflammatory stimuli. This can additionally cause inflammatory cell infiltration and cause progressive retinal damage [119]. Two major signaling pathways, such as NF-κB and JAK/STAT, play a key role in the development of autoimmune uveitis. They are responsible for regulating the expression of genes involved in inflammation and immune response [120]. Targeted therapies aimed at these pathways, such as JAK inhibitors and NF-κB modulators, enable precise suppression of excessive inflammatory activity, offering new treatment options for patients with autoimmune uveitis who do not respond to conventional medications [120]. The activation of the NF-κB pathway—triggered by pattern recognition receptors (PRRs) and pro-inflammatory cytokines like TNF-α and IL-1β—leads to the expression of numerous inflammatory molecules that fuel immune responses [121]. At the same time, the JAK/STAT signaling pathway, primarily stimulated by cytokines such as IL-6 and IFN-γ, supports the development and proliferation of Th1 and Th17 cells. These specific T cell subsets are known to drive inflammation and are heavily implicated in the development of autoimmune disorders, including autoimmune uveitis. While cytokines such as IL-6, low concentrations of TGF-β1, IL-1β, IL-23, and IL-21 drive Th17 differentiation, inhibitory effects are mediated by IL-27, IL-35, IL-2, IL-4, and IFN-γ. However, due to cytokine complexity and Th17 plasticity, further research is necessary to define precise markers and mechanisms for targeting pathogenic Th17 cells in autoimmune uveitis [122]. Peripheral tolerance—the ability of the immune system to avoid attacking the body’s own tissues—is largely maintained by dendritic cells (DCs). These cells help regulate immune responses by promoting the development of regulatory T cells or by selectively deactivating or eliminating potentially harmful T cells [123]. A key player in autoimmune inflammation of the eye is IL-17, mostly produced by Th17 cells. Both pro-inflammatory mechanisms play a key role in the complex interactions that drive autoimmune inflammation within the immune system. The DC is an important component of immune tolerance. It prevents autoimmunity by its ability to remove overactive T cells or by stimulating the growth of regulatory T cells [123]. One of the most significant pro-inflammatory factors is IL-17, which is primarily produced by Th17 cells. In addition to increasing vascular permeability and drawing neutrophils, IL-17 also directly causes tissue damage. One of the main factors influencing the course of the disease is TNF-α, which intensifies these inflammatory reactions even more [124]. To protect ocular tissue, regulatory T cells help to inhibit overreactive immune responses. In order to neutralize these pro-inflammatory signals. When this equilibrium is upset, ocular tissue may develop progressive damage and chronic inflammation. Macrophages are immune cells that aid in the treatment of inflammation and tissue repair [125]. New prospects for more focused and efficient treatments are presented by a better understanding of the immune mechanisms underlying autoimmune uveitis (Figure 6).

## 4. Modern Diagnostic Methods and Molecular Technologies

Advancements in molecular biology, genetic engineering, and omics technologies have created a new era of precision diagnostics, enabling the detailed analysis of biological processes at the level of DNA, RNA, proteins, and metabolites. These innovations have not only improved the accuracy and timelines of disease detection but also deepened our understanding of pathophysiological mechanisms [1,2,3]. Technologies such as next-generation sequencing (NGS), proteomics, metabolomics, molecular imaging, and liquid biopsy offer powerful tools for identifying diagnostic, prognostic, and predictive biomarkers [126,127]. Their application in ophthalmology has opened new avenues for personalized diagnostics and targeted therapies [4,128]. The integration of multi-omics data with imaging and artificial intelligence (AI)-based analytics is enabling the construction of comprehensive diagnostic models that capture the molecular complexity of ocular diseases [8]. Together, these molecular and imaging-based technologies are transforming ophthalmic diagnostics, offering unprecedented precision in disease detection, classification, and personalized treatment planning.

### 4.1. Genomics and Transcriptomics in Diagnostics

Genomic and transcriptomic technologies have revolutionized medical diagnostics by providing high—resolution molecular insights for disease etiology. Implementation of whole-genome sequencing (WGS) and whole-exome sequencing (WES) has been transformative in genomic diagnostics. WGS analyzes the entire genome, including coding and non-coding regions, while WES focuses solely on protein-coding regions (the exome), which is about 1–2% of the genome [126,129]. These methods allow for the detection of pathogenic variants underlying rare Mendelian disorders and polygenic conditions. However, interpretation of these methods has proved challenging due to the high burden of variants of unknown significance (VUS) [126,130]. Advancements in next-generation sequencing (NGS) have furthered clinical genomics through increased affordability, automation, and scalability. NGS, also known as high-throughput sequencing, enables the simultaneous sequencing of numerous DNA fragments, creating a quick and comprehensive analysis of the genome, especially compared to its former counterpart, Sanger sequencing [126]. NGS is particularly useful in diagnosing rare genetic disorders and tailoring oncologic therapies [126,127]. In ophthalmology, targeted NGS panels have substantially improved diagnostic yields in inherited retinal diseases (IRDs) such as Leber congenital amaurosis (LCA), retinitis pigmentosa (RP) and Stargardt disease, where causative variants in genes like RPE65, CEP290 and ABCA4 can now be directly identified [4,128,130]. Such insights guide eligibility for gene-specific treatments, including gene therapy for RPE65-associated LCA [127]. While targeted NGS is already implemented in clinical practice for IRDs, broader techniques such as WGS and WES are primarily used in specialized or research settings due to their complexity, cost, and interpretation challenges [131]. Liquid Biopsy is an emerging, non-invasive technique facilitated by NGS that detects circulating tumor DNA (ctDNA) or cell-free RNA (cfRNA) in peripheral fluids, including aqueous humor, vitreous humor, tears, and blood. Its application is expanding in ocular oncology, especially in monitoring uveal melanoma, where ctDNA detection allows for early relapse surveillance and molecular subtyping without requiring intraocular biopsy [4,126,129,132]. Additionally, cfRNA studies are being investigated for systemic diseases with ocular involvement, such as sarcoidosis-associated uveitis [132,133]. Despite promising results, liquid biopsy remains largely investigational in ophthalmology, with ongoing studies needed to validate its diagnostic performance and utility in routine care [133]. In parallel, RNA sequencing (RNA-seq) adds a crucial functional layer to diagnostics by illuminating transcriptional dysregulation. RNA-seq uses NGS to determine the exact sequence of nucleotides in the transcriptome, the complete set of RNA molecules in a cell or sample [133,134,135]. RNA-seq can be further divided into bulk and single-cell RNA sequencing (scRNA-seq). Bulk RNA-seq analyzes the average gene expression of a population of cells, while scRNA-seq measures gene expression in individual cells [134,135]. These tools enhance diagnostic yield in complex conditions with unclear genomic drivers. For instance, transcriptomic profiling has revealed unique expression variants in optic neuropathies, autoimmune uveitis, and transcriptome-disrupting syndromes affecting retinal cell subtypes [136,137,138,139]. In diabetic retinopathy, retinal scRNA-seq identifies glial and endothelial gene expression linked to inflammation and angiogenesis, refining biomarker discovery and disease staging [138,139,140,141]. However, both bulk and single-cell RNA-seq are currently used almost exclusively in research settings, and have not yet been validated for routine diagnostic application in ophthalmology [142]. More recently, long-read sequencing, optical genome mapping, and spatial transcriptomics have enabled the detection of structural variants, gene fusion, and tissue-specific transcriptional changes with unprecedented precision. These tools are especially impactful in complex ophthalmic cases, such as those with splice-site mutations or mitochondrial disorders, where standard exome analysis is uninformative [138]. For example, in diseases like Usher syndrome, long-read sequencing has clarified deep intronic mutations, including the USH2A, CEP290, and PCDH15 genes, that disrupt splicing [127]. Nevertheless, these methods remain in the preclinical or early translational stage and are not yet part of standard diagnostic workflows. Their broader adoption will require further validation in prospective clinical studies [143]. AI-powered bioinformatics has become essential in managing high-dimensional sequencing data. Machine learning algorithms enable the integration of multi-omics, enhancing disease classification and supporting biomarker discovery, particularly in oncology, neurodegeneration, and retinal disorders [139,144,145]. In ophthalmology, integrated AI models assist in the interpretation of RNA-seq and NGS results for conditions such as glaucoma, helping correlate transcriptomic alterations with early retinal ganglion degeneration [140]. Explainable AI (XAI) techniques provide further transparency in diagnostic models by identifying how specific omic features drive classification. This is especially helpful in personalized ophthalmology, where disease heterogeneity complicates clinical interpretation [141]. Moreover, AI-based transcriptome classifiers can now predict disease severity, subtype, and progression based on gene expression signatures. For example, in AMD, transcriptome classifiers help distinguish between dry and wet subtypes, supporting targeted therapeutic strategies [141,146]. When coupled with other omics layers, these classifiers contribute to the development of multi-omics digital twins, which simulate individualized disease trajectories and enable precision-guided diagnostic and therapeutic decisions [147]. Although AI-based interpretation tools are being developed, to our knowledge, there is currently no ophthalmic-specific AI model that has received clinical approval [148]. Despite these advances, challenges of high cost, limited access, and data complexity continue to limit broad clinical adoption. Further, ethical issues related to genetic privacy and incidental findings must be carefully managed [149,150]. Interpretation of novel or rare variants still requires expert curation and functional validation, especially in pediatric-onset ocular disorders [130]. Tools such as CRISPR-based diagnostics and AI-assisted genomic interpretation platforms are in development to detect ultra-sensitive mutations, and streamline diagnostics and enhance accessibility, respectively [151,152,153]. To maximize clinical impact, future efforts should focus on stratifying these technologies by readiness for implementation, clarifying which approaches are evidence-based and currently actionable, and which remain experimental. This distinction is crucial for informed clinical decision making and the responsible translation of molecular diagnostics into ophthalmic care (Table 1).

### 4.2. Proteomics and Metabolomics of the Eye

Proteomic and metabolomic analyses have proven instrumental in advancing molecular diagnostics, particularly in ophthalmology, where the eye’s compartmentalized structure allows for targeted sampling of biofluids such as aqueous humor, vitreous humor, tears, and plasma. These matrices offer rich molecular information tailored to specific ocular compartments and disease processes [144,145,147,149]. Proteomics is the identification and quantification of a set of proteins, the proteome. Metabolomics comprehensively analyzes metabolites, the intermediate or end products of metabolic reactions and regulators of metabolism, to describe and quantify the molecular composition of a sample, the metabolome [1,5]. Mass spectrometry (MS)-based proteomics, including liquid chromatography-MS (LC-MS) and high-resolution MS (HRMS), has identified protein signatures associated with key ocular diseases. In glaucoma, for instance, aqueous humor profiling has uncovered dysregulation in proteins such as ALD1A1, CRYAA, and clusterin, which are involved in oxidative stress, apoptosis, and neurodegeneration, reflecting retinal ganglion cell loss and aqueous outflow dysregulation [130,144]. Beyond aqueous humor proteins, metabolomics in glaucoma reveals disrupted energy metabolism—including aberrant levels of citrate, lactate, and pyruvate—that correlate with optic nerve degeneration, providing non-genetic functional markers for disease monitoring [144,145,150]. In DR, metabolomic profiling of vitreous and aqueous humor has consistently revealed markers linked to polyol pathway dysregulation, glycolytic imbalance, lipid peroxidation, and advanced glycation end-products. These markers not only stratify disease stages but also offer insights into microvascular leakage and neovascularization, with implications for early detection and therapeutic stratification [140,145]. AMD studies have identified metabolomic and proteomic alterations in lipid metabolism, mitochondrial dysfunction, and the complement cascade, distinguishing between dry and neovascular forms. Complement proteins, oxidative stress markers, and lipid mediators are being actively explored as early diagnostic and predictive biomarkers, particularly through plasma and aqueous humor-based liquid biopsy [141,144,152]. Multi-omics liquid biopsy approaches are being applied to complex ocular diseases such as autoimmune keratitis, thyroid eye disease, and ocular tumors, where systematic and local immune-metabolic dysregulation is seen. In thyroid eye disease, for example, circulating proteomic signatures have shown strong associations with inflammatory activity and tissue remodeling [145,146]. Tear-based omics have become the new frontiers in non-invasive diagnostics. In dry eye disease (DED), tear proteomics and metabolomics reveal consistent changes in interleukins (IL-1beta, IL-6), MMP-9, lipid mediators, and osmolarity-regulating metabolites, correlating with symptom severity and ocular surface damage [145,147]. Similarly, in herpes simplex keratitis (HSK), distinct viral-immune interaction markers have been identified through tear metabolomics, aiding early-stage diagnosis and response monitoring [149]. Targeted proteomic platforms, such as Olink proximity extension assays (PEA) and SomaScan aptamer arrays, are now enabling high-throughput, highly sensitive profiling from microliter-scale ocular samples. These platforms quantify cytokines, angiogenesis mediators, and neurodegeneration-related proteins, supporting their role in biomarker-guided clinical decision making [130,144]. From a technological standpoint, tools such as LC-MS/MS, NMR spectroscopy, and high-throughput metabolomics workflows have become standardized for ocular biomarker discovery [5,145]. AI-assisted data integration, including machine learning classifiers trained on multi-omics datasets, has produced diagnostic models with performance superior to conventional imaging in diseases like glaucoma, uveitis, and macular edema. These classifiers can stratify patients by disease subtype and predict progression trajectories with high precision [139,151]. Most proteomic and metabolomic diagnostics in ophthalmology are still in the translational or early phases of clinical validation, despite these advancements. Some of the limitations include the need for consistent protocols across centers and variations in sample collection and processing. Furthermore, these platforms are not commonly utilized in routine clinical practice due to their high cost and technical complexity. To validate biomarker reproducibility and clinical utility and to successfully incorporate these results into clinical decision-making processes, extensive longitudinal studies and multicenter trials are needed. Furthermore, when implementing multi-omics diagnostics, careful consideration should be given to ethical issues pertaining to data privacy and incidental findings (Table 2) [154,155,156,157].

### 4.3. Molecular Imaging and Biomarkers

Molecular imaging technologies provide spatial and functional context to diagnostics by visualizing the distribution of biomolecules within intact tissue architecture. These automations are crucial for disease localization, biomarker validation and spatial omics integration [7,8]. This is especially important in histopathologically complex tissues like the retina, choroid, and optic nerve head, where spatial heterogeneity impacts diagnosis and prognosis [150]. Among the foundational tools, Optical Coherence Tomography (OCT) is a cornerstone in retinal diagnostics, offering micrometer-resolution cross-sectional imaging of retinal layers. It is routinely used in the assessment and monitoring of AMD, DR, and glaucoma, where structural changes such as retinal edema, macular thinning, or ganglion cell loss are early indicators of disease [6,7,139]. Fluorescence imaging, including fundus autofluorescence (FAF) and fluorescein angiography (FA), is instrumental in detecting lipofuscin accumulation (e.g., In Stargardt disease), retinal pigment epithelium (RPE) dysfunction, and vascular leakage in retinal vascular disorders like diabetic macular edema and central retinal vein occlusion [6,152]. Furthermore, MRI with contrast agents enables visualization beyond the retina, which is useful for optic nerve pathology, orbital tumors, or inflammatory diseases such as thyroid eye disease and optic neuritis, where systemic correlations are essential [7,146]. Continually, Positron Emission Tomography (PET), though more often used in systemic oncology, is gaining ground in ocular diagnostics, especially for ocular melanoma and autoimmune thyroid orbitopathy by using tracers that monitor glucose metabolism or immune activation [8,133]. One of the most powerful tools is matrix-assisted laser desorption/ionization imaging mass spectrometry (MALDI-MSI), which enables label-free, spatially resolved detection of lipids, proteins, and metabolites. In AMD, Maldi-MSI has revealed distinct molecular gradients of oxidized phospholipids and complement-associated peptides in retinal pigment epithelium and Bruch’s membrane, distinguishing early dry AMD from neovascular subtypes [141,152]. In retinopathy of prematurity (ROP) and choroidal melanoma, MALDI-MSI has enabled molecular subtyping based on lipid dysregulation and tumor-specific proteomic signatures, providing histology-guided diagnostic stratification and guiding treatment decisions [133,144,152]. Similarly, spatial transcriptomics, another key innovation, enables high-resolution gene expression mapping within retinal architecture. It has delineated VEGFA, ANGPT2, and IL1B upregulation in localized retinal zones of diabetic retinopathy and HLA-DR/IFNG-positive immune clusters in non-infectious uveitis, improving our understanding of microenvironmental disease heterogeneity [134,150]. Imaged-based biomarkers are quantifiable structural or functional features that correlate with disease activity or progression. Firstly, retinal thickness, measured by OCT, is a critical marker in macular edema, AMD, and inherited retinal dystrophies and is commonly used to monitor therapeutic response to anti-VEGF agents or corticosteroids [6,7,128]. Secondly, FAF evaluates RPE stress and degeneration in retinitis pigmentosa, Stargardt disease, and AMD, demonstrating accumulation of toxic fluorophores like A2E with detection of abnormal signs [128,152]. Tertiarily, Capillary nonperfusion and neovascularization are central in proliferative diabetic retinopathy and neovascular AMD. Visualization of this via OCT angiography (OCTA) guides the timely initiation or escalation of anti-angiogenic therapy [6,7,140].

Finally, Ganglion cell-inner plexiform layer (GCIPL) thinning, identified on OCT, is emerging as an early neurodegenerative biomarker in glaucoma and optic neuropathies, often even preceding visual field loss [6,139]. Next-generation workflows increasingly combine molecular imaging with omics layers to create multimodal diagnostic pipelines. In wet AMD and retinal vein occlusion, imaging of VEGF-targeted molecular probes or nanobody-based fluorophores is under development to enable real-time angiogenesis visualization, potentially allowing dynamic, individualized adjustment of anti-VEGF therapy intervals [7,151]. Furthermore, in thyroid eye disease, imaging of extraocular muscle hypertrophy is now being correlated with serum proteomic markers of inflammation and fibrosis [146]. In glaucoma, structural OCT metrics, including retinal nerve fiber layer thickness, have been paired with aqueous humor proteomics, identifying markers such as ALDH1A1 and clusterin, revealing novel molecular pathways of neurodegeneration [139,140]. Thus, molecular imaging tools bridge omics data with in vivo pathology, allowing validation of candidate biomarkers and direct visualization of molecular processes with disease [7,151]. The field of molecular imaging is rapidly evolving towards precision diagnostics through multi-omics imaging integration and novel imaging agents. The development of AI-driven imaging analysis is now standard in OCT and fundus image interpretation, with deep learning models capable of detecting AMD, diabetic macular edema, and glaucoma, providing expert-level accuracy [10,151,158,159]. These models are continually integrating transcriptomic and proteomic profiles, enabling composite digital biomarkers and personalized risk models [139,151,158]. Additionally, theranostics is a rapidly advancing domain where molecular imaging agents also deliver therapeutic payloads. For instance, nanoformulations targeting inflammatory cytokines or angiogenic mediators are being trialed for uveitis and AMD, offering concurrent diagnosis and treatment [7,8]. Moreover, molecular contrast agents designed to target oxidative stress, complement activation, or apoptosis pathways are under development for application in retinal degenerative and autoimmune eye diseases [7,140]. These innovations are also being tested as surrogate endpoints in clinical trials, accelerating drug development in diseases like DR, inherited retinal disorders, and ocular tumors [7,127,133]. Further trials and testing will allow the invention of precision diagnostics through molecular imaging coupled with omics technologies and AI-based analytics, offering a transformative framework for understanding, diagnosing, and treating ophthalmic diseases. Through high-resolution, disease-specific visualization and integration with molecular data, it is redefining the paradigm of ocular diagnostics [7,151,158]. It is important to keep in mind that, despite their enormous potential for diagnosis and treatment, many of the omics-integrated and molecular imaging techniques described above are still in the experimental or early clinical research stages. Imaging modalities such as OCT, OCTA, FA, FAF, and MRI are commonly used and well-established in clinical settings for the diagnosis and monitoring of diseases like AMD, glaucoma, and diabetic retinopathy [160]. However, at the moment, theranostic molecular probes, spatial transcriptomics, and MALDI-SI are not used in routine clinical practice [161]. These methods are typically employed in preclinical investigations or early-phase clinical trials and require additional validation for clinical efficacy, safety, and cost-effectiveness [160,161]. Similarly, AI-based picture interpretation models are rapidly being used in clinical settings, but their integration with omics data to build composite biomarkers is still under development [162]. As a result, distinguishing between clinically established diagnostic methods and those that are still being investigated is critical for providing an informed perspective on their current and future clinical utility (Table 3 and Table 4).

## 5. Innovative Therapeutic Approaches

### 5.1. Gene Therapies

**Figure 7:**
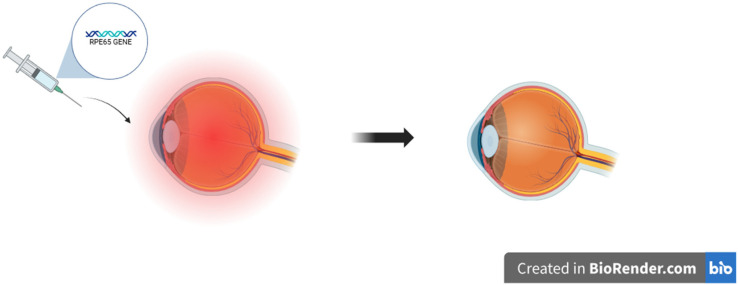
Gene Therapies. Therapeutic vectors are delivered to the eye through different administration routes, including intracameral injection, intrastromal injection, or subconjunctival injection. The aim is to provide a functional copy of the RPE65 gene to the retinal pigment epithelium (RPE) cells [163,164,165,166,167,168,169,170,171,172,173,174].

The use of gene therapies in ophthalmology is one of the most rapidly evolving areas of modern medicine. Due to particular properties of the eye, such as small size and immunologically privileged position, it is an optimal location for the implementation of molecular strategies. A gene augmentation is a procedure that includes providing cells with a functional copy of a gene to cover up missing or malfunctioning genes [163,164]. This approach, which is commonly used to treat autosomal and X-linked recessive retinal abnormalities, is safe and well-established [163]. An FDA-approved adeno-associated virus 2-based (AAV2) treatment for individuals with biallelic RPE65 mutations, voretigene neparvovec (Luxturna), is a noteworthy achievement. During the therapy, vectors are delivered by intracameral, intrastromal, subconjunctival, or even by topical application and restore the visual cycle by providing a functional copy of the RPE65 gene (Figure 7) [164,165,166]. Gene therapies are becoming increasingly effective treatments for a range of eye diseases. Gene therapies might be exceptionally helpful in retinal and optic nerve neuropathies involving inherited retinal dystrophies (IRDs). By precisely delivering therapeutic genes to retinal cells, viral gene therapy aids in the restoration of photoreceptor function [166,167,168]. Gene therapy for glaucoma aims to preserve retinal ganglion cells (RGCs) and lower intraocular pressure (IOP). Genes that regulate aqueous humor outflow and lower IOP, such as MMP-3, miR-146a, and PGF2α, have been successfully delivered via AAV and provide a satisfying effect [166,169,170]. Clinical studies are also being conducted to reduce IOP using siRNA-based treatments that target β2-adrenergic receptors. Moreover, applying CRISPR/Cas9 gene editing using RGC-specific promoters may possibly have neuroprotective features [166,169,171,172]. Due to the cornea’s easy accessibility and immunological privilege, gene therapy is making progress in managing corneal disorders. Preclinical models are being used to evaluate several delivery techniques for the treatment of keratitis, corneal fibrosis, scarring, dystrophies, and neovascularization [166,173,174].

### 5.2. Cellular and Regenerative Therapies

In the treatment of ocular diseases, cellular and regenerative therapies offer a revolutionary strategy that aims to repair and regenerate damaged tissues in addition to stopping the disease’s progression [175]. One of these treatment methods may be a stem cell therapy used in retinal diseases. Clinical research demonstrates that RPE cells made from pluripotent stem cells are safe and tolerable when used to treat retinal dystrophies. Cell-based therapy presents a double action, the first path is focused on the replacement of damaged cells with new retinal cells made from stem cells that can fuse with the host tissue and restore retinal function. The second path aims at the release of trophic factors that support the regenerative effect [176,177,178]. Research showed that this therapy may be useful in the treatment of age-related macular degeneration (AMD) and retinitis pigmentosa (RP) [176,178]. Another type of cell-based therapy used in ophthalmology might be corneal stem cell therapy. Studies presented that the transplantation of limbal epithelial stem cells (LESCs) and corneal stromal stem cells (CSSCs) may be a successful method of corneal surface and corneal stromal regeneration [179]. Limbal epithelial cell transplantation (CLET) using LESCs either from human embryonic stem cells (hESCs) or induced pluripotent stem cells (iPSCs) has become a clinical success, particularly when the amniotic membrane serves as a supporting substrate [179,180]. According to the research, CLET is safe and efficient in almost 80% of instances, long-term corneal healing was observed [179]. At this point, the only LESC treatment authorized in Europe for certain limbal stem cell deficiency instances is Holoclar. In the meantime, CSSCs show promising perspective but are still in the preclinical stage; further research is required to fully understand their potential [179,181,182]. Recently, new treatment methods using induced pluripotent stem cells (iPSC) demonstrate effectiveness in managing numerous monogenic diseases [183]. As mentioned above, IPSCs may be used in macula-related diseases, but it has also been discovered that IPSCs present therapeutic features in Retinal ganglion cell (RGC) degeneration, reducing the progression of inflammation and supporting the recovery of vision. Research showed that there is also a potential approach to treating optic nerve disorders, but the lack of unambiguous positive results demands further investigation [184,185]. However long-term graft survival is still a crucial factor, even if recent data shows that stem cell-based ocular therapies are safe and effective in the short to medium term. Graft survival rates in limbal stem cell transplantation range greatly, from roughly 50% to 100% throughout follow-ups of 16 to 49 months; this variation is probably driven by immunosuppressive regimes and rejection risk [186]. Furthermore, it has not yet been determined that undifferentiated stem cells, especially pluripotent varieties like iPSCs, have the potential to develop into tumors (teratomas). Despite the encouraging short- and medium-term safety of stem cell-based ocular therapies, the possibility of carcinogenesis is still a significant worry, especially with pluripotent stem cells like iPSCs, which have been demonstrated to generate teratomas even in tiny residual numbers of undifferentiated cells [187,188]. Tumors can be easily produced by residual undifferentiated iPSCs; in animal experiments, teratoma development can be induced by as little as 10^5^ cells [189]. The requirement for extremely sensitive purification techniques and quality control is highlighted by the hazards associated with the reprogramming process and residual pluripotent factors, which can arise from genomic instability or oncogene involvement [190]. Thus, to guarantee both long-term therapeutic effectiveness and patient safety, strict purification procedures in conjunction with ongoing preclinical and clinical monitoring are crucial [191].

### 5.3. Biological and Molecular Therapies

Nowadays, molecular and biological therapeutics are essential for treating ocular conditions, especially those affecting the retina [192]. Among the most important developments in this area is the use of monoclonal antibodies, which can provide highly accurate pathogenic protein targeting. Monoclonal antibodies might be used in inflammatory diseases that affect the eyes. Choe et al. reported the positive effect of deploying anti-TNF-α monoclonal antibodies in a group of pediatric patients with uveitis. After implementing adalimumab for a year, 21 out of 22 eyes (95.5%) presented no anterior chamber inflammation [193]. Patients with uveitis who were treated with anti-TNF-α antibodies presented significantly fewer side effects than those who were using corticosteroids. Moreover, research indicates that with appropriate administration of monoclonal antibodies, reliance on corticosteroids can be considerably decreased to prevent recurrent uveitis [194]. Another group of medications frequently used in ophthalmology is vascular endothelial growth factor (VEGF) inhibitors. Deploying anti-VEGF in a group of patients with AMD revolutionized the course of treatment. The suppression of ocular neovascularization induced by VEGF, which is one of the fundamental pathogeneses of AMD, significantly inhibits the progression of the disease [195,196]. Ricci et al. presented that the usage of Ranibizumab—a completely humanized monoclonal antibody fragment that attaches itself to several VEGF-A isoforms, effectively prevents the formation of new vessels and modifies the course of AMD [195]. VEGF inhibitors are also an efficient tool in the fight against proliferative diabetic retinopathy (PDR). PDR is the primary cause of blindness in Americans of working age, accounting for between 12,000 and 24,000 new cases annually. High blood sugar level contributes to the damage of retinal blood vessels, reducing their lumen and leading to hypoxia. Moreover, high blood sugar level increases the process of neovascularization [197,198]. Gross et al. presented a randomized clinical trial that confirms a significant improvement and reduction in vessel damage in a 5-year period of PDR [197]. VEGF inhibitors contribute to reduced visual field loss and to the decreased occurrence of diabetic macular edema (DME) that impairs vision. Furthermore, severe vision loss, or major PDR consequences such as macular traction retinal detachments, neovascular glaucoma or iris neovascularization were reported less frequently [197,199]. VEGF inhibitors are also an effective therapy for retinopathy of prematurity. The blockade of VEGF production and new blood vessel formation successfully avoids the unfavorable outcomes of prematurity retinopathy [200,201]. Stahl et al. Reported about 85.5% treatment effectiveness of using intravitreal aflibercept [201]. What is also important is that Chang et al. reported that while bevacizumab is used instead of laser treatment for Zone I prematurity retinopathy, the risk of required retreatment is much lower- 67% lower [202]. Despite the established advantages, an extensive number of patients have unsatisfactory responses to anti-VEGF therapy, which are known as tachyphylactic, refractory, or recalcitrant reactions. These responses are frequently characterized by residual effusion or recurrence even after several injections [203]. Tachyphylaxis after repeated ranibizumab injections, upregulation of alternative pro-angiogenic pathways like PDGF in response to VEGF suppression, involvement of macrophage-mediated inflammation, and dysregulation of cholesterol are some of the mechanisms linked to this resistance. Arteriolar choroidal neovascularization (CNV) and resistance are also caused by these mechanisms [204,205,206]. The use of combination approaches has shown promise in addressing these issues. For example, concurrent anti-VEGF and anti-PDGF therapy has been shown to provide a significant additional benefit over monotherapy in neovascular AMD, and new strategies that target macrophage cholesterol efflux have been shown to overcome arteriolar CNV and resistance in preclinical models [206]. Furthermore, in patients with partial response, moving to more recent bispecific medicines like faricimab (which targets both VEGF-A and Ang-2) has improved anatomical outcomes and decreased treatment burden in practical settings [207].

### 5.4. Targeted Therapies

**Figure 8:**
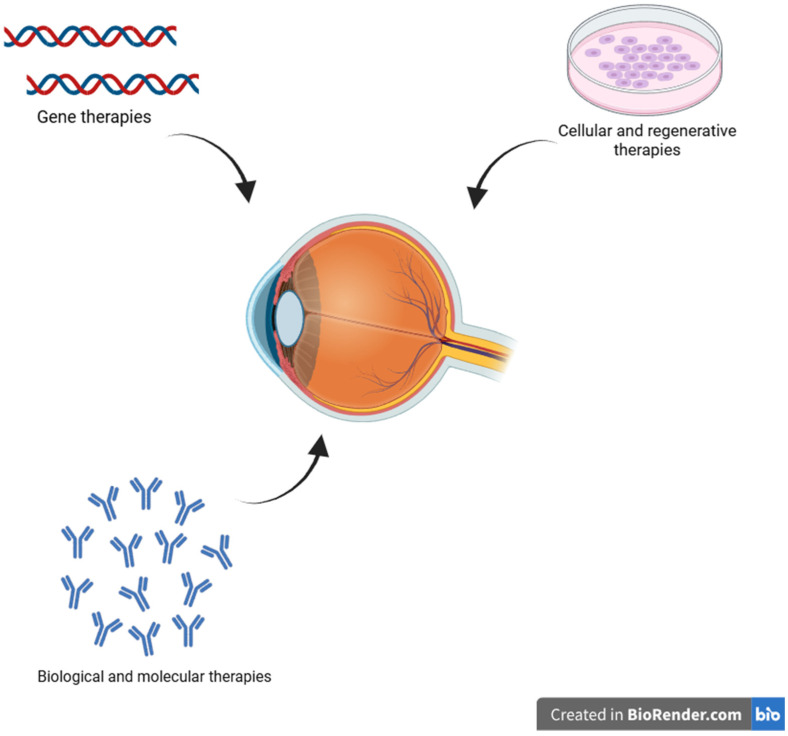
Modern therapeutic approaches in the treatment of ophthalmological diseases. The graphic shows potential innovative therapeutic methods that might be deployed in the treatment of different eye diseases.

In ophthalmology, targeted therapies are a crucial component of modern treatment for vascular, inflammatory, and retinal eye disorders due to their ability to allow for precision action on particular molecular targets. VEGF inhibitors such as faricimab, ranibizumab, aflibercept, and brolucizumab are injected intravitreally (into the vitreous body of the eye). They are one of the most commonly implemented targeted therapies in this field [195,208,209]. High concentrations of the active ingredient can reach the posterior portion of the eye, especially the retina and choroid, thanks to the procedure’s usual outpatient setting [208,210]. These therapies are mostly used to treat retinopathy of prematurity, central or branch retinal vein occlusion, DME, and AMD [195,196,197,199,210]. Additionally, patients suffering from non-infectious uveitis are increasingly receiving immunomodulatory treatments, particularly monoclonal antibodies that block pro-inflammatory cytokines like adalimumab (anti-TNF-α monoclonal antibody) [193]. In contrast to anti-VEGF inhibitors, these medications are generally administered intravenously or subcutaneously as a component of systemic immunosuppressive treatment [211,212]. Therapies that target particular signaling pathways, like Notch and MAPK, are also gaining interest. These therapies are primarily in preclinical or early clinical stages and are intended for the treatment of glaucoma and degenerative retinal diseases (Table 5), (Figure 8) [213,214,215].

## 6. Future Research Directions and Clinical Perspectives: Toward Personalized Ophthalmic Therapies

The era of patient-specific precision medicine has been ushered in by molecular biology, advanced imaging, gene and cell therapies, and artificial intelligence [216]. Revolutionary discoveries have been made, such as anti-VEGF therapies and biologics for uveitis, especially for chronic, progressive, and genetic eye diseases [217]. Understanding cellular heterogeneity in both healthy and diseased ocular tissues is possible thanks to the presence of new technologies, such as single-cell transcriptomics and proteomics, which offer previously unseen solutions [218]. For example, in experimental autoimmune uveitis, single-cell RNA sequencing has identified specific inflammatory microglial subpopulations as potential therapeutic targets [219]. Predicting individual therapeutic responses and reducing adverse events may be made possible by integrating genomic data with cytokine biomarkers like IL-6 or TNF-α [220]. This strategy supports adaptive dosing, as illustrated in other inflammatory conditions, though ophthalmic trials are still nascent [221]. The approval of voretigene neparvovec (Luxturna) for RPE65-mediated inherited retinal dystrophy marked a milestone in ocular gene therapy [222]. Phase 3 trials demonstrated significant improvements in multi-luminance mobility (~1.6 light-level gain; *p* = 0.0013) and no serious immunologic effects [222]. Treatment of retinal diseases such as AMD and diabetic retinopathy is possible thanks to immune evasion techniques and optimization of vector delivery [223]. Early detection, prognosis, and monitoring are revolutionized by deep learning applied to OCT, fundus, and OCTA images. Meta-analyses show pooled high diagnostic performance: DR detection in OCT/fundus studies yielding sensitivity/specificity > 0.90 [224]. DME on OCT achieves 96% sensitivity and 99% specificity, while CNNs identify AMD with AUC ~0.93–0.98 [225]. However, lack of explainability and dataset heterogeneity remain barriers to clinical adoption. To improve the accuracy of delivery, safety, and treatment frequency, innovations such as robot-assisted injection guided by real-time OCT imaging and sustained-release formulations have been developed [226]. The high costs of new treatments and changing reimbursement patterns are an inherent challenge for physicians [227]. Ethical concerns about long-term safety, vector immunity, and genomic privacy; regulatory barriers for gene-based and personalized therapies; and the requirement for extensive, multicenter trials with a range of demographics [228]. Cost–benefit analysis and stringent postmarketing monitoring are crucial. A new approach to ophthalmic care that emphasizes biological individuality is being heralded by the combination of cellular-level profiling, AI-driven diagnostics, and customized interventions [229]. Coordinated, multidisciplinary research, innovative regulatory frameworks, and stakeholder collaboration—from basic science to clinical implementation and health policy—are necessary to realize this vision.

## 7. Current Limitations and Challenges

Molecular imaging and precision therapies are emerging therapeutic approaches that require an assessment of their limitations, which can impact treatment outcomes [230]. Technological limitations, such as imaging resolution and tracer specificity, can limit diagnostic accuracy [231]. The high costs associated with purchasing or leasing advanced imaging equipment and the use of targeted therapies represent a significant barrier to their widespread adoption [232]. Patient variability, including varying biological responses, is another key factor that can reduce the predictability and effectiveness of these therapies. Where data are sparse, this gap must be clearly defined to ensure transparency [233]. Addressing these limitations is essential to establishing realistic expectations and guiding future research.

## 8. Synergistic Interactions and Multimodal Therapeutic Strategies

Understanding how precision drugs and molecular imaging may work in tandem to improve patient outcomes is essential to incorporating them into clinical practice [234]. Both molecular imaging methods and targeted drugs can be beneficial in improving therapy monitoring and facilitating the early detection of resistance [235]. For example, combining immunotherapy with PET imaging has shown promise in improving treatment response assessment and patient selection [236]. This review enhances translational relevance and provides a clearer roadmap for future clinical applications by presenting real-world examples of this multimodal synergy.

## 9. Conclusions

In summary, advanced analytics, technologies, and molecular imaging are transforming ophthalmology, improving diagnosis and treatment. Accurate disease localization, biomarker validation, and identification of retinal and optic nerve heterogeneity are enabled by high-resolution, non-invasive techniques such as OCT, OCTA, MALDI-MSI, and spatial transcriptomics. These methods are essential for identifying biomarkers that guide personalized treatment, as well as for early detection and monitoring of common eye diseases such as AMD, diabetic retinopathy, glaucoma, and uveitis. For hereditary and degenerative diseases, gene therapies such as AAV-based vectors and cell-based regenerative techniques using iPSCs or LESCs hold great promise. Furthermore, monoclonal antibodies and anti-VEGF drugs are examples of biologics that consistently improve the course of inflammatory and vascular diseases. Artificial intelligence-based imaging processes and emerging theranostic techniques are further integrating diagnostics with individually tailored interventions. Despite these advances, challenges remain related to delivery, safety, access, and regulation. To provide precise eye care tailored to each patient’s molecular and structural profile, future success depends on interdisciplinary collaboration and continuous innovation.

## Figures and Tables

**Table 1:** Summary of Genomic and Transcriptomic Technologies and Their Applications in Ophthalmic Diagnostics.

Technology	Description	Key Features	Applications in Ophthalmology
**Whole Genome Sequencing (WGS)** [126,129]	Sequencing of the entire genome, coding and non-coding regions	Comprehensive, detects all variants	Rare Mendelian disorders, polygenic diseases
**Whole Exome Sequencing (WES)** [126,128,129,131]	Sequencing protein-coding regions (~1–2% of genome)	Focused on exons, cost-effective	Diagnosing inherited retinal diseases (IRDs)
**Next-Generation Sequencing (NGS)** [126,127,131]	High-throughput sequencing of multiple DNA fragments simultaneously	Affordable, automated, scalable	Rare genetic disorders, oncology, targeted gene panels
**Targeted NGS Panels** [4,128,131]	Sequencing specific gene sets	Higher diagnostic yield, faster results	IRDs like LCA, RP, Stargardt disease
**Liquid Biopsy** [4,126,128,133]	Detects circulating tumor DNA (ctDNA) or cell-free RNA (cfRNA) in fluids	Non-invasive, dynamic monitoring	Uveal melanoma, systemic ocular diseases
**RNA Sequencing (RNA-seq)** [133,134,135,138,139]	Sequencing of transcriptome (all RNA molecules)	Bulk and single-cell options, functional insight	Transcriptional dysregulation in retinal diseases
**Single-Cell RNA-seq (scRNA-seq)** [134,135,138,139,140,141]	Gene expression at the individual cell level	High resolution, detects cell heterogeneity	Diabetic retinopathy, autoimmune uveitis
**Long-Read Sequencing** [127,138]	Sequencing long DNA fragments	Detects structural variants, splice mutations	Usher syndrome, mitochondrial disorders
**Optical Genome Map-ping** [138]	Visualizes structural variants in the genome	High precision, structural variant detection	Complex inherited retinal diseases
**Spatial Transcriptomics** [138,150]	Maps gene expression spatially within tissue	Tissue-specific transcriptional profiling	Retinal microenvironment heterogeneity
**AI-Powered Bioinformatics** [139,140,141,144,145,146,147]	Machine learning for multi-omics data integration	Enhances classification, biomarker discovery	Glaucoma, AMD, retinal degeneration
**CRISPR-based Diagnostics** [151,152,153]	Nucleic acid detection with CRISPR nucleases	Ultra-sensitive mutation detection	Emerging genomic diagnostic tool

**Table 2:** Proteomic and Metabolomic Findings and Technologies Used in Key Ophthalmic diseases.

Ophthalmic Disease	Key Molecular Findings	Technologies Used
Glaucoma [130,144,145,150]	Dysregulated proteins: ALD1A1, CRYAA, clusterin; metabolites: citrate, lactate, pyruvate	LC-MS, HRMS, LC-MS/MS
Diabetic Retinopathy (DR) [140,145]	Markers of polyol pathway dysregulation, glycolytic imbalance, lipid peroxidation	Metabolomic profiling of aqueous/vitreous humor
Age-Related Macular Degeneration (AMD) [141,144,152]	Alterations in lipid metabolism, mitochondrial dysfunction, and complement cascade proteins	Plasma and aqueous humor proteomics/metabolomics
Autoimmune/Tumor Diseases [145,146]	Proteomic signatures linked to inflammation and tissue remodeling	Circulating proteomics (e.g., Olink PEA)
Dry Eye Disease (DED) [145,147]	Elevated IL-1β, IL-6, MMP-9, lipid mediators, osmolarity metabolites	Tear proteomics and metabolomics
Herpes simplex Keratitis (HSK) [149]	Viral-immune interaction markers	Tear metabolomics

**Table 3:** Molecular Imaging Technologies and Their Applications in Ophthalmology.

Imaging Technology	Ophthalmic Applications	Key Biomarkers/Findings	Notes and Emerging Uses
Optical Coherence Tomography (OCT) [6,7,139,151,158]	Retinal layer imaging in AMD, DR, glaucoma	Retinal thickness, macular edema, GCIPL thinning	Standard for disease monitoring and therapy response
Fundus Autofluorescence (FAF) [6,128,152]	RPE dysfunction, Stargardt disease, retinitis pigmentosa	Lipofuscin (A2E) accumulation, RPE stress	Detects early degeneration, toxicity markers
Fluorescein Angiography (FA) [6,152]	Retinal vascular leakage in diabetic macular edema, CRVO	Vascular leakage, capillary nonperfusion, neovascularization	Guides anti-angiogenic therapy
Magnetic Resonance Imaging (MRI) [7,146]	Optic nerve pathology, orbital tumors, inflammatory diseases	Visualization beyond retina, optic neuritis, thyroid eye disease	Systemic correlation and orbital imaging
Positron Emission Tomography (PET) [8,133]	Ocular melanoma, autoimmune thyroid orbitopathy	Glucose metabolism, immune activation	Expanding use in ocular oncology and inflammatory orbitopathies
MALDI Imaging Mass Spectrometry (MALDI-MSI) [133,141,144,152]	AMD, ROP, choroidal melanoma	Oxidized phospholipids, complement peptides, lipid dysregulation	Label-free, spatially resolved molecular detection
Spatial Transcriptomics [134,150]	Diabetic retinopathy, non-infectious uveitis	Localized VEGF-A, ANGPT2, IL1B, immune clusters	Maps gene expression within retinal architecture
OCT Angiography (OCTA) [6,7,140]	Proliferative diabetic retinopathy, neovascular AMD	Capillary perfusion, neovascularization	Non-invasive vascular imaging
AI-Driven Imaging Analysis [10,139,151,158]	AMD, diabetic macular edema, glaucoma	Automated detection and classification of structural changes	Integration with omics for composite digital biomarkers
Theranostic Molecular Imaging [7,8,151]	Uveitis, AMD	Targeted nanobody-fluorophores, inflammatory and angiogenic markers	Concurrent diagnosis and therapy delivery

**Table 4:** Summary of Diseases, Pathophysiological Mechanisms, Imaging Techniques, and Therapies.

Disease	Main Pathophysiological Mechanisms	Imaging Techniques	Therapies
AMD	Lipid peroxidation, mitochondrial dysfunction, complement activation	OCT, FAF, MALDI-MSI	Anti-VEGF therapy, iPSC-derived cells, gene therapy
Glaucoma	Oxidative stress, apoptosis of RGCs, neurodegeneration	OCT, OCTA, GCIPL analysis	Neuroprotection, IOP-lowering drugs, gene therapy
Diabetic Retinopathy (DR)	Polyol pathway dysregulation, angiogenesis, advanced glycation end-products	OCT, FA, OCTA	Anti-VEGF therapy, targeted molecular therapy, gene therapy
DME	Vascular leakage, inflammation, cytokine upregulation	OCT, OCTA	Anti-VEGF agents, corticosteroids
Keratitis	Immune-mediated inflammation, corneal neovascularization	OCT, molecular imaging	Biologic therapies, tear-based diagnostics, gene therapy
Dry Eye Disease (DED)	Hyperosmolarity, inflammatory cytokines, immune cell infiltration	Tear proteomics, anterior segment OCT	Omics-based diagnostics, epithelial regeneration
Retinopathy of Prematurity	Pathological angiogenesis due to oxygen fluctuations	FA, OCTA	Anti-VEGF agents

**Table 5:** Technologies, Clinical Stage, and Target Diseases.

Technology/Platform	Clinical Stage	Target Diseases	Data Type
OCT/OCTA	Established in clinical routine	AMD, glaucoma, DR, DME	Structural imaging
FA/FAF	Widely used in clinical ophthalmology	DR, AMD, Stargardt disease	Fluorescence-based imaging
MALDI-MSI	Preclinical/early clinical research	AMD, uveal melanoma	Spatial proteomics
Spatial transcriptomics	Early-stage studies	DR, uveitis	Spatial gene expression
Proteomics	Translational/early clinical	Glaucoma, DED, autoimmune diseases	Protein biomarkers
Metabolomics	Translationa	AMD, DR, HSK, DED	Small-molecule metabolites
AI in Imaging	Partially deployed in clinics	AMD, DR, glaucoma	Imaging diagnostics
Theranostics	Preclinical/Phase I	Uveitis, AMD	Imaging + therapy
Gene therapie	Approved (RPE65), others in trials	IRDs, glaucoma	Genetic correction
Cell therapies	Partially approved (e.g., Holoclar)	AMD, RP, corneal disorders	Regenerative medicine
Biologic therapies (e.g., anti-VEGF, anti-TNF)	Approved	AMD, DR, PDR, uveitis	Targeted molecular therapy

## Data Availability

Not applicable.

## Abbreviations

AAV2	approved adeno-associated virus 2
ACAID	Anterior Chamber-Associated Immune Deviation
AGE	advanced glycation and end-products
AI	artificial intelligence
AMD	age-related macular degeneration
ANGPT2	angiopoietin-2
BM	Bruch’s membrane
BRB	blood-retina barrier
CAT	catalase
cfRNA	cell-free RNA
CLET	Limbal epithelial cell transplantation
CNV	choroidal neovascularization
CRISPR	clustered regularly interspaced short palindromic repeats
CRVO	central retinal vein occlusion
CSSCs	corneal stromal stem cells
ctDNA	circulating tumor DNA
DAF	decay-accelerating factor
DCs	dendritic cells
DED	dry eye disease
DME	diabetic macular edema
DR	diabetic retinopathy
FA	fluorescein angiography
FAF	fundus autofluorescence
GCIPL	ganglion cell-inner plexiform layer
GPX	glutathione peroxidase
GWAS	genome-wide association study
HIF-1α	transcription factor 1 alpha
HLA-DR/IFNG	Human Leukocyte Antigen—DR isotype/Interferon gamma
hESCs	human embryonic stem cells
HSK	herpes simplex keratitis
HRMS	high-resolution mass spectrometry
IL-1beta/IL1B	Interleukin 1beta
IL-6	Interleukin 6
IOP	intraocular pressure
iPSC	induced pluripotent stem cell
iPSCs	induced pluripotent stem cells
IRD	inherited retinal dystrophies
IRDs	inherited retinal diseases
LC-MS	liquid chromatography-mass spectrometry
LCA	Leber congenital amaurosis
LESCs	limbal epithelial stem cells
MAC	membrane attack complex
MALDI-MSI	matrix-assisted laser desorption/ionization imaging mass spectrometry
MCP	membrane cofactor protein
MMP-9	Matrix metalloproteinase-9
MOMP	membrane permeabilization
MRI	Magnetic resonance imaging
MS	mass spectrometry
NF-κB	Nuclear Factor kappa-light-chain-enhancer of activated B cells
NGS	next-generation sequencing
NMR	nuclear magnetic resonance spectroscopy
O-GlcNAcylation	O-linked β-N-acetylglucosaminylation
OCT	Optical Coherence Tomography
OCTA	OCT angiography
PDR	proliferative diabetic retinopathy
PEA	proximity extension assays
PET	Positron Emission Tomography
PKC	protein kinase C
PRDXs	peroxiredoxins
PRRs	pattern recognition receptors
RGC	retinal ganglion cell
RGCs	retinal ganglion cells
RNA	ribonucleic acid
RNA-seq	RNA sequencing
ROP	retinopathy of prematurity
RP	retinitis pigmentosa
RPE	retinal pigment epithelium
scRNA-seq	single-cell RNA sequencing
SOD	superoxide dismutase
TM	trabecular meshwork
UV	ultraviolet
VEGF	vascular endothelial growth factor
VEGF-A	vascular endothelial growth factor A
WES	whole-exome sequencing
WGS	whole-genome sequencing
WHO	World Health Organization
XAI	Explainable AI
